# Radiation used for head and neck cancer increases virulence in *Candida tropicalis* isolated from a cancer patient

**DOI:** 10.1186/s12879-017-2879-6

**Published:** 2017-12-20

**Authors:** Eliane Martins da Silva, Elaine Sciuniti Benites Mansano, Ellen Sayuri Miazima, Francielle Abigail Vilugron Rodrigues, Luzmarina Hernandes, Terezinha Inez Estivalet Svidzinski

**Affiliations:** 1 Department of Medical Mycology, State University of Maringá, Av. Colombo, 5760, C.P, Maringá, PR 87020-900 Brazil; 2 Department of Histopathology, State University of Maringá, Av. Colombo, 5760, C.P. 87020900, Maringá, Paraná Brazil

**Keywords:** Gamma radiation, *Candida tropicalis*, Infection, Biofilm, Virulence

## Abstract

**Background:**

Studies have shown that radiation from radiotherapy increases the yeast colonization of patients. However it is not clear, if such radiation alters the yeast itself. The aim of the present study was therefore to report the direct impact of gamma radiation on *Candida tropicalis.*

**Methods:**

*C. tropicalis* was obtained from a patient with a carcinoma, a suspension of this yeast containing 2.0 × 10^3^ colony forming units per milliliter was prepared. It was submitted to gamma radiation dosage similar to that used in the treatment of head and neck cancer. After a cumulative dose of 7200 cGy some virulence attributes of *C. tropicalis*, including macro and micromorphological characteristics, adhesion and biofilm abilities, murine experimental infection and phagocytosis resistance were evaluated on irradiated and non-irradiated yeasts.

**Results:**

After irradiation the colony morphology of the yeast was altered from a ring format to a smooth appearance in most colonies. Scanning electron microscopy revealed notable differences in the structures of both these colonies and the yeast cells, with the loss of pseudohyphae following irradiation and an increase in extracellular matrix production. The adherence and biofilm production of the yeast was greater following irradiation, both in terms of the number of yeasts and total biomass production on several abiotic surfaces and TR146 cells. The phagocytic index of the irradiated yeasts was not statistically different; however, the presence of cellular debris was detected in the kidneys of infected animals. Mice infected with irradiated yeasts developed an infection at the site of the yeast inoculation, although systemic infection was unchanged.

**Conclusions:**

Our findings show for the first time that *C. tropicalis,* one of the most important yeasts from colonization, which cause fatal candidemia in cancer patients, is affected by gamma irradiation, with changes to its virulence profile.

## Background

Head and neck cancers account for more than 500,000 cases worldwide every year, and the numbers of such illnesses are increasing [1]. According to a review, *Candida* species are indirectly associated with the carcinogenesis of some forms of cancer, such as Head and Neck Squamous Cell Carcinoma (HNSCC) and Oral Squamous Cell Carcinoma (OSCC). Candidemia is also a common invasive fungal infection (IFI) among cancer patients [1–3]. In many cases, the yeasts that cause such infections are the result of the normal colonization of the patient following radiotherapy. Studies have found that once radiotherapy begins, the rate of yeast colonization of patient increases, and can reach to 74%. In addition, has been estimated that approximately 26% of colonized patients develop localized infections, while 37% develop candidemia, the most severe clinical manifestation [2–4].

Among species of the *Candida* genus, *C. albicans* has received the most clinical attention, including studies on the pathophysiology of IFI. However, besides the global increase of these infections, reports have shown an increase in the prevalence of *Candida* non-*C. albicans* (CNCA), such as *C. tropicalis*, *C. parapsilosis* and *C. glabrata* [1]*. C. tropicalis* is among the most important of them, especially in tropical countries [5, 6]. In India, this species represents 64% of all yeasts identified over a 3.5-year period [7], and it is also the most isolated CNCA in Brazilian hospitals, where it accounts for around 20% of candidemia cases [8]. *C. tropicalis* is considered an important agent of candidemia, especially in neoplasia patients [9, 10]. This is partly due to the virulence characteristics of this species, such as its high adhesion capacity and ability to form biofilm on surfaces [11]. *C. tropicalis* from clinical isolates naturally exhibits highly diversified macromorphological variations known as phenotypic switching [12, 13], an ability associated with cell damage in epithelial tissue. Although this virulence attribute has been observed in animal model [14], it is not known if the same occurs in humans. It is also not known if yeast exposed to radiation suffer changes in their putative virulence factors.

The influence of external factors such as exposure to the radiation used in treatment, which seems to affect yeasts present in colonization, should also be considered. Other such factors include filamentation, antifungal resistance and faster growth [15–19]. Gamma radiation therapy is one of the most commonly used cancer treatments, especially for patients with head and neck cancer [20, 21]. Among other adverse effects, this therapy significantly reduces the production of saliva and salivary pH, allowing greater yeast colonization [3]. A secondary effect of radiation treatment is the development of mucositis due to the rupture of the primary barrier of the mucosa [22], which can act as a gateway for the development of infectious processes by these yeasts.

Candidemia in cancer patients is generally attributed to host debility, and little is known about the direct impact of radiation on yeasts. A pioneering study by Mendling and Haller, 1977 [15] found that yeasts irradiated in isolation exhibited alterations in cellular morphology, antimycotic sensitivity and pathogenicity. In 1988, Procop et al. [16] identified morphological and physiological changes in *Candida* species*,* such as accelerated growth and the increased pseudohyphae and blastopore production. Meanwhile, Dambroso et al. [17] identified changes in the virulence of *C. albicans* isolated from patients during radiotherapy treatment. Other studies have shown that a single application of gamma radiation can cause alterations in human pathogenic yeasts, such as inhibiting the in vivo growth of *Candida* and *Cryptococcus* [18]. Also was reported an increase of adhesion of yeasts on catheter surfaces, as well as their resistance to antifungal agents [19]. Despite these studies on the effects of gamma radiation on *Candida* yeasts, to the best of our knowledge the effects of a radiation scheme equivalent to those employed in human therapy, including dose and time of exposure, have not been evaluated.

The aim of the present study was therefore to evaluate the possible impact of a complete gamma radiation, scheme similar to those used in the treatment of head and neck cancer, applied directly on yeast, evaluating some virulence parameters of a *C. tropicalis* isolate, which was obtained from a patient with laryngeal carcinoma.

## Methods

### Microorganism

A clinical isolate of *Candida tropicalis* was obtained from a patient diagnosed with head and neck cancer prior to cancer treatment. The experimental protocol was approved by the Standing Committee on Ethics in Research with Human Beings of the State University of Maringá (COPEP 254/2006). Oral lavage samples were collected and cultivated on Sabouraud Dextrose Agar (SDA) (Difco, Sparks, Maryland, USA), and the yeasts grown were identified by classical methods.

### Radiotherapy treatment simulation

A radiotherapy treatment scheme recommended for head and neck cancer was imitated and applied to a pure culture of *C. tropicalis,* which had been isolated soon after the diagnosis of neoplasia. A suspension of yeast containing 2.0 × 10^3^ colony forming units per milliliter (CFU/mL) was grown in 15-ml Falcon conical tubes containing Sabouraud Dextrose Broth (SDB) (Difco, Sparks, Maryland, USA). The tubes were exposed to radiation used in telecobalt therapy applied in the same manner as it is with patients. An irradiation area of 9 cm × 26 cm on right and left sides and column angles of 270° and 90° were used. The distance between the target (*C. tropicalis* cultures) and the equipment source was 80 cm; the depth of the target was 6.5 cm; and the depth dose percentage was 72.8%. The irradiation rate was 50.93 cGy/min for 2m25s in 40 applications of 180 cGy/day for each side, totaling 7200 cGy. The cultures were irradiated once a day, five days a week, for eight weeks with a daily fraction dose of 180 cGy. Following irradiation, the yeasts were frozen at −80 °C in SDB with 50% glycerol. A control culture was prepared in the same way and maintained under equal conditions, including of transport and temperature, ensuring that gamma irradiation was the only difference between the yeasts. The control yeasts were described as “non-irradiated” while the yeasts exposed to radiation were referred to as “irradiated”.

### Parameters evaluated in *Candida tropicalis* before and after irradiation

#### Micro and macromorphology characteristics

Macromorphology was examined following the reactivation of the irradiated yeast in 2 mL of Yeast Peptone Dextrose (YPD) liquid medium and incubated at 25 °C for 24 h. The inoculum was adjusted to a density of approximately 300 CFU/mL, uniformly seeded on plates containing YPD agar medium and maintained at room temperature for 96 h. The colonies were photographed under a digital microscope. The ultrastructure of the colony was analyzed by scanning electron microscopy (SEM) after lyophilization in accordance with Moralez et al. [14].

#### Adhesion and biofilm forming ability

The assay was performed in accordance with Porman et al. [13] with some modifications: the yeasts were reactivated overnight in YPD broth at 35 °C and cultured for 24 h in YPD agar. Some colonies were resuspended in 3 ml of YPD broth and incubated at 35 °C for one night. The inoculum was adjusted in YPD broth at 2 OD_600_, and 1 ml of this preparation was transferred to a 24-well polystyrene plate, which was incubated at 25 °C for two hours (to evaluate adhesion) and 24 h (biofilm), without agitation. Each well was washed three times with 1 mL of sterile water and was scraped to remove the adherent cells. The cells were then resuspended in 1 mL of sterile water and biofilm density was determined by OD_600_ spectrometry. To verify the number of CFU the samples were prepared in the same manner as described above and resuspended in 1 mL of water. A quantity of 10 μL was then collected and placed in SDA and cultured at 35 °C for 24 h to count the number of colonies. Adhesion and biofilm were also measured by crystal violet staining. After being washed three times, the plates were dried for 45 min and then stained with 0.4% violet crystal in water (385 μL per well) for 45 min. Each well was washed three times with 1 mL of sterile water and bleached with 700 μL of 95% ethanol. A volume of 100 μL from each well was then transferred to a 96-well plate. Optical density was determined by spectrophotometry at OD_595_. The experiment was performed in triplicate and in three independent assays.

The same method as for the adhesion on plates was used to evaluate the adhesion and formation of biofilm on silicon, except 5 mm × 5 mm sections of silicone were added and left in the yeast suspension in 1 mL cryotubes. After incubation for two hours at 25 °C the silicon coupons were transferred to new sterile cryotubes, were washed three times and resuspended in 1 mL of water. An ultrasound was used to detach the adhered yeasts. To determine the number of adhered yeasts 10uL was collected and plated. The density of the adhered cells was determined by OD600 spectrometry.

Adhesion on the TR146 cell culture was performed according to Murciano et al. [23]*.* Briefly, the TR146 cells were cultivated in a chamber with 5% CO_2_ in 24-well microplates following the culture instructions for these cells. After obtaining the monolayer, irradiated and non-irradiated *C. tropicalis* were added to the wells, and were then incubated for 90 min at 37 °C. After incubation, the non-adherent yeasts were removed by aspiration and the plates were scraped to release the adhered yeasts, which were inoculated in plates containing SDA medium and incubated at 30 °C for 24 h. The plates were observed under a microscope to ensure that all the cells had been removed. The resulting colonies were then counted. The experiments were carried out in triplicate and repeated on three different occasions.

#### Murine experimental infection

All the procedures were performed according to the regulations of the institutional Ethics Committee for animal experimentation of the State University of Maringá (approval number. CEP 056/2013, 07/11/2013). Systemic infection was performed in accordance with Xie, Jing et al. [24] with some modifications: eight-week-old male and female Swiss mice (*Mus musculus*) were infected intravenously through the caudal vein with 100 μL of PBS containing ~ 1 × 10^7^ yeasts/mL. The animals were divided into two groups: those infected with non-irradiated *C. tropicalis* (before gamma radiation) and those infected with irradiated yeasts (after gamma radiation). Two males and two females per yeast type were used. The animals were sacrificed on the third day following infection. The right kidney of each animal was collected, weighed and macerated with 2 mL of PBS. The maceration product was diluted to 1:100, plated in SDA, and incubated at 35 °C for 24 h. Two independent assays were performed. For histological study the left kidney was collected and fixed in 4% paraformaldehyde for 24 h. Once fixed, the kidneys were kept in 70^o^ alcohol, which was replaced every 24 h until processing for inclusion in paraffin. Semi-serial sections with a thickness of 5 mm were stained with silver and counter-stained light green according to the technique described by Gomori-Grocott. The preparations were observed and photographed using a Nikon Eclipse 80i trinocular optical microscope with a Nikon camera (DSFi1C), coupled to a computer using Nis-Element software (Tokyo, Japan).

#### Phagocytosis resistance

In vitro phagocytosis assay was performed following the isolation of peritoneal macrophages from mice in accordance with Ray and Dittel [25]. Briefly, the macrophages were counted and transferred ~ 5 × 10^5^ to 24-well polystyrene culture microplates containing coverslips, and incubated at 37 °C, 5% CO2, for two hours. The cultures were washed once, resuspended in 500 μL of RPMI and incubated at 37 °C, 5% CO2 for 24 h. Each well was washed with PBS to remove the unbound cells and 500 μL of new RPMI was added. *C. tropicalis* was cultivated in YPD broth at 35 °C overnight before the suspension was washed and diluted at a ratio of five yeasts per phagocyte. It was then added to the wells containing the coverslips with the macrophages attached and incubated for two hours at 35 °C. Each well was washed once with PBS and the coverslips were removed and stained with haematoxylin eosin (H&E) (fast panoptic staining LB; Laborclin, Paraná, Brazil). After overnight drying, each coverslip was affixed to slides and phagocytic activity was determined by the phagocytic index using light microscopy. A total of 200 phagocytes per slide were counted and the experiment was performed in duplicate in two independent assays.

#### Statistical analysis

Statistical analysis was performed using the Prism 6.0 software package (GraphPad, San Diego, CA, USA) and significance was determined using the Mann-Whitney pair-wise test, the unpaired t-test and the Wilcoxon non-parametric test or ANOVA datasets. Values of *p* < 0.05 were considered statistically significant.

## Results

Comparative assays between the irradiated and non-irradiated yeasts showed that gamma irradiation altered some parameters of *C. tropicalis,* such as its morphological characteristics, local infection capacity, adhesion on abiotic and biotic surfaces, biofilm formation and phagocytic index.

**Fig. 1:**
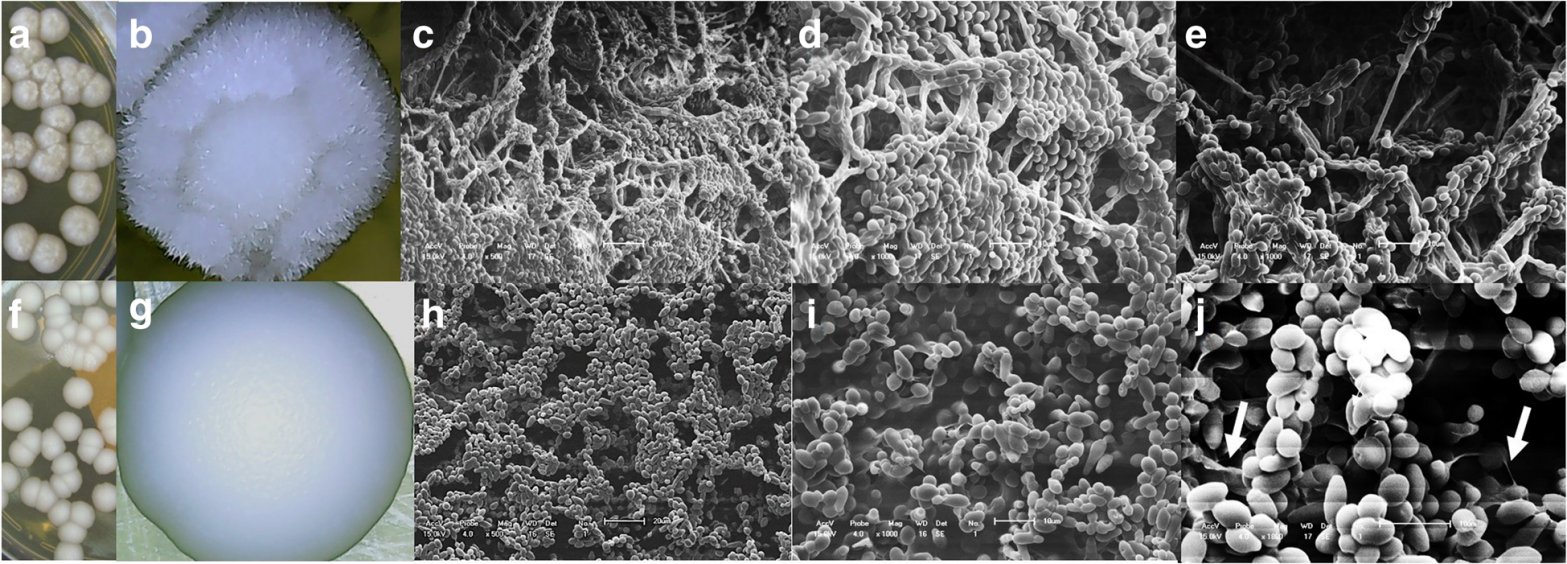
*Candida tropicalis* clinically isolated from the oral mucosa of a patient with laryngeal carcinoma prior to any cancer or antifungal treatment. The top line shows images of non-irradiated yeast (NI) and the lower line shows images of yeast after receiving a cumulative dose of 7200 cGy (**i**). Parental phenotypes of colonies of NI yeast with ring morphology (**a**, **b**) and parental phenotypes of I yeast with smooth morphology (**f**, **g**). Scanning electron microscopy showing the microstructure of colonies after 96 h of cultivation in YPD agar at 25 °C: surface of parental colony of NI yeasts with ring morphology (**c**) and surface of parental colony of I yeasts with smooth morphology (**h**). Ultrastructure of parental colony of NI yeasts, ring morphology, where blastoconidia and pseudohyphae are observed (**d**, **e**) and parental colony of I yeasts, smooth morphology, absence of pseudohyphae and thick extracellular matrix indicated by white arrows (**i**, **j**)

Non-irradiated yeast colonies had a parental ring format and an aerial mycelium (Fig. 1a-b), while the aerial mycelium disappeared completely in the irradiated yeasts, which had a smooth appearance (Fig. 1f-g). This characteristic was maintained following ten consecutive repetitions. SEM revealed significant changes in the ultrastructure of the colonies and the morphology of the yeasts. The colonies of the non-irradiated yeasts had a rough appearance (Fig. 1c), consisting of blastoconidia and pseudohyphae (Fig. 1d-e). After irradiation there was an evident change in the composition of the colony (Fig. 1h), and a total absence of pseudohyphae (Fig. 1i-j).

**Fig. 2:**
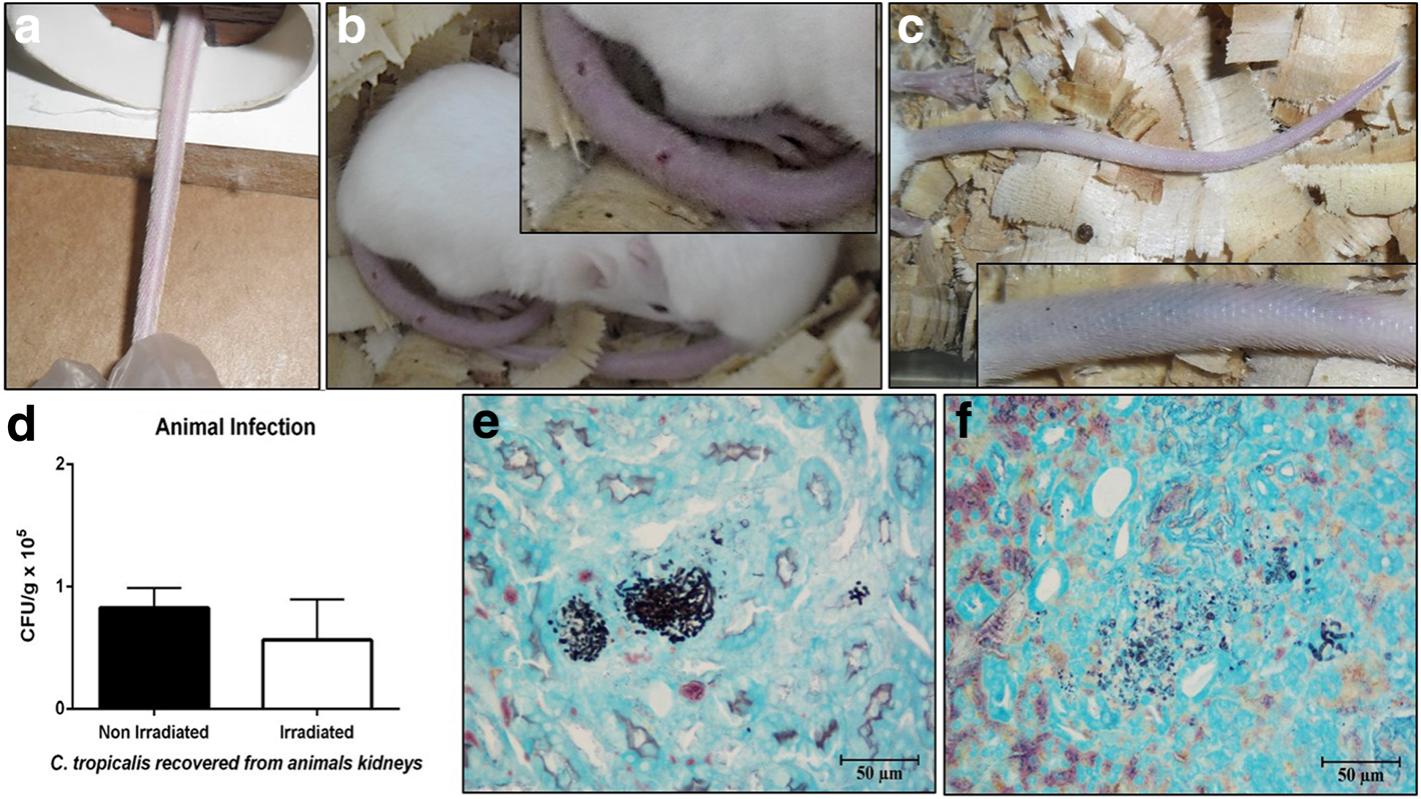
Swiss mice (*Mus musculus*) infected via the caudal vein with clinical isolate of *Candida tropicalis* after receiving a cumulative dose of 7200 cGy. (**a**) Tail of no infected animals (before yeast inoculation). The tail of animals infected with irradiated yeasts after 72 h of infection showed a visible inflammatory process, with reddish color, lethargic animals and piloerection (**b**). Cyanotic tail after 24 h of infection with irradiated yeasts (**c**). Graph representing yeasts recovered from the kidneys, difference not statistically significant. Data collected from two independent experiments. Standard deviation represented by bars (**d**). Kidney infected with non-irradiated (**e**) and irradiated yeasts (**f**) stained with silver by the Gomori-Groccot technique

The irradiated yeasts provoked more clinical signals than the non-irradiated yeasts in the infected mice. The animals infected by irradiated *C. tropicalis* were collected from the bottom of the cage and were lethargic with bristly hairs. In contrast, the behavior of the animals infected by non-irradiated yeasts did not differ from the uninfected mice (control group). The number of yeasts recovered from the kidneys of the infected animals did not statistically differ between the groups with yeast either before and after irradiation (Fig. 2d), a finding confirmed through histopathology (Fig. 2e-f). Only the group infected with irradiated yeasts exhibited signs of infection and cyanosis at the application site (Fig. 2b-c).

**Fig. 3:**
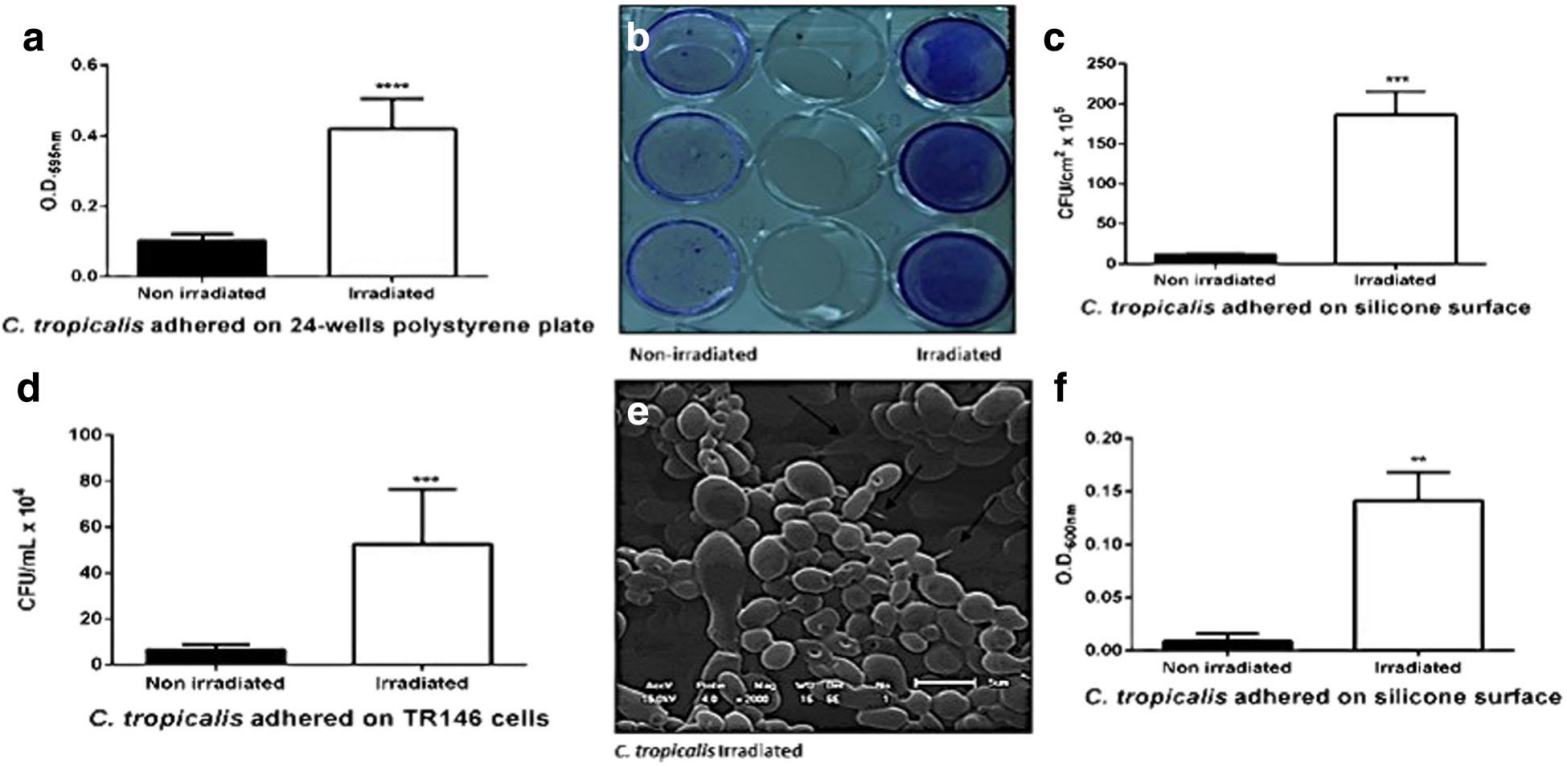
Adhesion assay with non-irradiated and irradiated *Candida tropicalis* on different surfaces and methodologies. Crystal Violet staining showed a significant increase of biomass (**a**), with the difference visible to the naked eye in a 24-well microplate (**b**), with the thick extracellular matrix (arrows) visible in SEM (**e**). Adhesion capacity on TR146 line cells increased significantly after irradiation (**d**). Irradiation provoked a significant increase in adhesion on silicon in both the number of adhered yeast cells (**c**) and the adhesion density (**f**). Data from three independent experiments. Standard deviation represented by bars. (*) indicates significant difference between samples (non-irradiated and irradiated)

In polystyrene plate adhesion assay, *C. tropicalis* produced a significant increase in biomass, stained with Crystal Violet, after radiation (Fig. 3a-b). The SEM arrows in SEM (Fig. 3e) indicate a large quantity of extracellular matrix. The same alteration was observed on the silicon surface, both in the number of adhered colonies and the total biomass without staining (Fig. 3c-f). Adherence of the yeasts to the TR146 tumor cell lines was then tested, and high adhesion capacity was again identified, which increased significantly after irradiation (Fig. 3d).

**Fig. 4:**
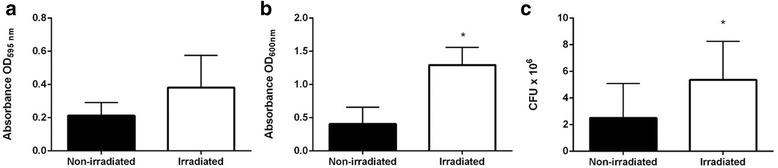
Biofilm forming capacity of *Candida tropicalis* (non-irradiated and irradiated). Crystal Violet staining suggests an increase of biomass although the difference was not significant (**a**); however there was a significant increase in biofilm density (**b**) and in the number of yeasts (**c**) after 24 h in a 24-well culture plate. Data from three independent experiments. Standard deviation represented by bars. (*) Indicates significant difference in biofilm formation between samples

The biofilm assay revealed that there was no significant difference in biomass stained with Crystal Violet (Fig. 4a). However, as was observed in the silicone surface adhesion test, there was a significant increase in biofilm density in OD_600_ and the number of CFU (*p* > 0.05) (Fig. 4b-c).

**Fig. 5:**
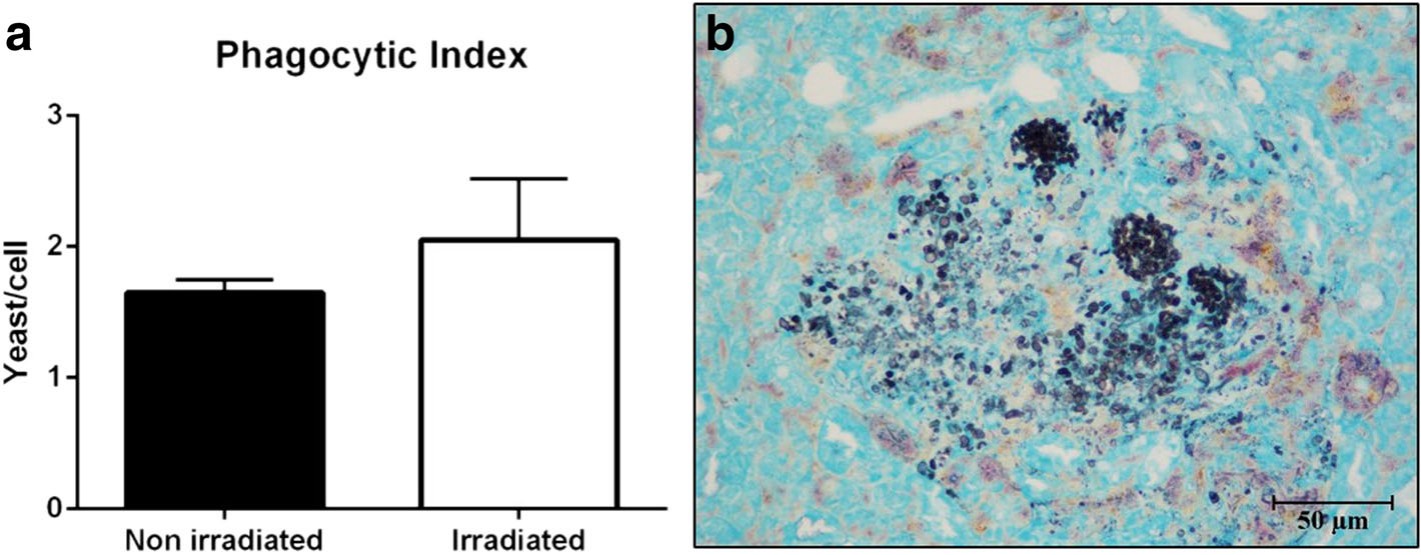
Phagocytic index of clinical isolate of *Candida tropicalis* before and after receiving a cumulative dose of 7200 cGy; while there was an increase in phagocytosis rate after irradiation, the difference was not significant (**a**). Gomori-Groccot silver staining revealed the cellular remains of yeasts in the kidneys of animals infected with irradiated yeasts, suggesting incomplete phagocytosis (**b**). Data from two independent experiments. Standard deviation represented by bars

Phagocytosis assay revealed a slight increase in the phagocytic index after irradiation, although the difference was not statistically significant (Fig. 5a). This data is corroborated by the histopathological analysis of the kidneys of the animals, where yeast cell debris was observed, evidenced by silver, as well as incomplete phagocytosis close to the yeast agglomerates (Fig. 5b).

## Discussion

The alterations on virulence factors in human pathogenic yeasts following gamma irradiation have been previously studied [16–18, 20] and colony morphology analysis have been carried out in spontaneous switching studies of *C. tropicalis* [11–13]. No studies showing the effects of irradiation on this species following a scheme equivalent to those used in human therapy were found, however. The results of the present study also identified morphological diversity. Fig. 1 exclusively illustrates the majority of the morphologies found in a total of five thousand colonies analyzed from each group (non-irradiated and irradiated *C. tropicalis*). Macromorphological and ultrastructural analysis (SEM) of the parental colonies (Fig. 1d-e and i-j) indicated that alterations occurred in the shape of the yeast cells, demonstrating a loss of filamentation after irradiation. This was surprising, as it was hoped that radiation would increase filamentation, as occurs with *C. albicans*. According to Zhang et al., (2016) [26] there are multiple interconnected signaling pathways involved in the regulation of filamentation in *C. tropicalis,* which may be responsible for the divergent features among different *Candida* species.

SEM revealed that the extracellular matrix of yeasts with a smooth morphological pattern became much thicker after irradiation (Fig. 1j). This type of morphological pattern has already been observed by França et al. [12], who described the spontaneous presence of the matrix as a maintenance factor of the morphology of the colony.

*C. tropicalis* usually adheres to and proliferates effectively on the epithelial tissue, and can invade and cause cellular damage to the oral epithelium [27]. In addition, changes in the morphology of the colony have been adequately described for this species, and have been related to changes at a cellular level [13]. It is therefore likely that, as well as its tissue invasion capacity, the cellular alterations provoked by gamma radiation had an impact on yeast virulence, as all the animals in the current study infected with the irradiated yeasts after 24 h, both male and female, exhibited swelling and redness in the tail at the inoculum application site (Fig. 2b-c). This did not occur in any of the animals infected by non-irradiated yeasts. These local inflammation signals have never previously been observed in our laboratorial experience.

It was also observed that animals infected with irradiated yeasts displayed lethargic behavior, weakness and piloerection. Considering that mice use their tails for balance and locomotion, it can be supposed that the tail pain caused by the inflammatory process was so intense that it made it impossible for the animals to feed, making them weaker. Animals infected by non-irradiated yeasts, meanwhile, exhibited normal feeding and locomotion behavior, similar to non-infected mice.

Curiously, the systemic infection was similar in both animal groups (mice infected by irradiated or non-irradiated *C. tropicalis*) as the numbers of yeast CFU recovered in the kidneys of the animals were not statistically significant (Fig. 2d). These data seem to be related to reduced filamentation ability after irradiation (Fig. 1), as bud-to-hyphae transition is considered a key event in the invasion of and adhesion to other cells and tissues in *C. tropicalis* [28]. Additionally, the data suggest that irradiated yeasts strongly adhere to the epithelium of the blood vessel soon after inoculation, causing signs and symptoms of apparent local infection, but not resulting in increased dissemination. These results reinforce our previous study [da Silva et al., 2017] where we also found that gamma radiation provoked changes in the virulence factors of the *C. tropicalis* strain. However, we have observed different findings regarding the ability to cause systemic infection, as in the aforementioned study irradiated *C. tropicalis* ATCC 750 provoked a significant increase in experimental murine systemic infection, accompanied by larger pseudo-hyphae production. Different behavior, meanwhile, was observed in terms of reduced adhesion on the TR146 cell line, with no changes in adherence ability and biofilm production and greater resistance to phagocytosis [29]. The possible explication for these discrepancies is that the previous study used irradiation from a reference strain, whereas the present work isolated *C. tropicalis* from a patient with cancer and employed the same irradiation conditions.

We strongly believe that the yeast which colonized the mucosa of the patient was more evasive of the host defense ability even before cancer treatment, and irradiation simply potentialized these virulence attributes. This hypothesis is supported by greater adherence and biofilm production ability on various surfaces and a lesser impact against phagocytic action than was suffered by the reference strain (Silva et al., 2017) [29], which was maintained in vitro for a longer period.

The absence of conclusive methodologies to confirm that gamma radiation increased the ability of *C. tropicalis* to cause localized infection in a mice model is a limitation of this study. Additional studies involving cellular interaction-host immune response experiments could strengthen this conclusion. Nevertheless, the hypothesis that the irradiated clinical isolate of *C. tropicalis* caused greater localized than disseminated infection can be explained by the high adherence capacity of *C. tropicalis* and its ability to form biofilm, as observed by other authors [11, 13, 27], as well as by the notable results observed in the polystyrene plate adhesion test (2 h), which revealed that this capacity increased significantly following irradiation, and which was confirmed in the present study under different methods (Fig. 3). Another relevant fact that reinforces this hypothesis is the result obtained by phagocytosis assay, which although not statistically significant, suggested that *C. tropicalis* became more susceptible to phagocytosis following irradiation (Fig. 4a). This observation is complemented by the histopathology of the kidneys, which revealed the presence of cell debris close to agglomerates of intact yeasts, suggesting incomplete phagocytic activity (Fig. 4b). All these findings are in accordance with the lower number of CFU of irradiated yeasts recovered in the kidneys (Fig. 2d), and probably explain why irradiation did not provoke an increase in the dissemination capacity of *C. tropicalis* in mice in the present study.

Despite its new findings, the present study has limitations. Firstly, the evidence of localized infection is weak, as discussed above. Another potential limitation is the use of just one strain. While a greater number of clinical isolates are needed to confirm our hypothesis, this is the first contribution to this line of study and we believe the results presented here are sufficiently strong to support our conclusions.

## Conclusions

The gamma radiation applied in this study caused morphological and physiological alterations in *C. tropicalis* related to the increased virulence of this clinical isolate. Although disseminated infection did not improve, the ability to cause localized infection appeared to be more efficient. Considering that this yeast is part of the microbiota of patients who undergo radiotherapy, this can be considered an additional risk of infection. These laboratory findings, shown here for the first time, suggest that radiation not only damages the mucous membranes of the patient, but also acts on the yeasts present at the irradiated site.

## Abbreviations

CFU/mL: Colony forming units per milliliter
CNCA: *Candida* non-*C. albicans*
HNSCC: Head and neck squamous cell carcinoma
IFI: Invasive fungal infection
OSCC: Oral squamous cell carcinoma
SDA: Sabouraud Dextrose Agar
SDB: Sabouraud Dextrose Broth
SEM: Scanning electron microscopy
YPD: Yeast Peptone Dextrose

## References

[1] Alibek K, Kakpenova A, Baiken Y (2013). Role of infectious agents in the carcinogenesis of brain and head and neck cancers. Infect Agent Cancer.

[2] Pinel B, Cassou-Mounat T, Bensadoun RJ (2012). Oropharyngeal candidiasis and radiotherapy. Cancer Radiother.

[3] Mañas A, Cerezo L, de la Torre A, García M, Alburquerque H, Ludeña B (2012). Epidemiology and prevalence of oropharyngeal candidiasis in Spanish patients with head and neck tumors undergoing radiotherapy treatment alone or in combination with chemotherapy. Clin Transl Oncol.

[4] Kurosawa M, Yonezumi M, Hashino S, Tanaka J, Nishio M, Kaneda M (2012). Epidemiology and treatment outcome of invasive fungal infections in patients with hematological malignancies. Int J Hematol.

[5] Chai LY, Denning DW, Warn (2010). *candida tropicalis* in human disease. Crit Rev Microbiol.

[6] Kothavade RJ, Kura MM, Valand AG, Panthaki MH (2010). *Candida tropicalis*: its prevalence, pathogenicity and increasing resistance to fluconazole. J Med Microbiol.

[7] Jain N, Mathur P, Misra MC, Behera B, Xess I, Sharma SP (2012). Rapid identification of yeast isolates from clinical specimens in critically ill trauma ICU patients. J Lab Physicians.

[8] Colombo AL, Guimarães T, Sukienik T, Pasqualotto AC, Andreotti R, Queiroz-Telles F (2014). Prognostic factors and historical trends in the epidemiology of candidemia in critically ill patients: an analysis of five multicenter studies sequentially conducted over a 9-year period. Intensive Care Med.

[9] Nucci M, Queiroz-Telles F, Alvarado-Matute T, Tiraboschi IN, Cortes J, Zurita J (2013). Epidemiology of candidemia in Latin America: a laboratory-based survey. PLoS One.

[10] Doi AM, Pignatari ACC, Edmond MB, Marra AR, Camargo LF, Siqueira RA (2016). Epidemiology and microbiologic characterization of nosocomial candidemia from a Brazilian National Surveillance Program. PLoS One.

[11] Negri M, Martins M, Henriques M, Svidzinski TI, Azeredo J, Oliveira R (2010). Examination of potential virulence factors of *Candida tropicalis* clinical isolates from hospitalized patients. Mycopathologia.

[12] França EJG, Andrade CGTJ, Furlaneto-Maia L, Serpa R, Oliveira MT, Quesada RM, Furlaneto MC (2011). Ultrastructural architecture of colonies of different morphologies and biofilm produced by phenotypic switching of *Candida tropicalis*. Micron.

[13] Porman AM, Hirakawa MP, Jones SK, Wang N, Bennett RJ (2013). MTL-independent phenotypic switching in *Candida tropicalis* and a dual role for *Wor1* in regulating switching and filamentation. PLoS Genet.

[14] Moralez AT, Perini HF, Furlaneto-Maia L, Almeida RS, Panagio LA, Furlaneto MC (2016). Phenotypic switching of *Candida tropicalis* is associated with cell damage in epithelial cells and virulence in *Galleria mellonella* model. Virulence.

[15] Mendling W, Haller I (1977). The effect of therapeutic doses of gamma radiation on *Candida albicans* cells *in vitro*. Geburtshilfe Frauenheilkd.

[16] Procop GW, Anderson-Davis H, Volz PA (1988). Cobalt 60 radiation and growth of *Candida* species. Mycoses.

[17] Dambroso D, Svidzinski TIE, Svidzinski AE, Dalalio MMO, Moliterno RA (2009). Radiotherapy effect on frequency of *Candida* spp. and on virulence of *C. albicans* isolated from the oral cavity of head and neck cancer patients. Rev Cienc Farm Básica Apl.

[18] Ben-Yosef R, Zeira M, Polacheck I (2005). The effect of radiation therapy on fungal growth: results of *in vitro* and *in vivo* studies. J Inf Secur.

[19] Farrag HA, A-Karam El-Din A, Mohamed-El-Sayed ZG, Abdel-Latifissa S, Kamal MM (2015). Microbial colonization of irradiated pathogenic yeast to catheter surfaces: relationship between adherence, cell surface hydrophobicity, biofilm formation and antifungal susceptibility. A scanning electron microscope analysis. Int J Radiat Biol.

[20] Geara FB, Sanguineti G, Tucker SL, Garden AS, Ang KK, Morrison WH (1997). Carcinoma of the nasopharynx treated by radiotherapy alone: determinants of distant metastasis and survival. Radiotherapy and oncology: journal of the European Society for Therapeutic. Radiol Oncol.

[21] VanderWalde NA, Fleming M, Weiss J, Chera BS (2013). Treatment of older patients with head and neck cancer: a review. Oncologist.

[22] Ahmadi A (2012). Potential prevention: Aloe Vera mouthwash may reduce radiation-induced oral mucositis in head and neck cancer patients. Chin J Integr Med.

[23] Murciano C, Moyes DL, Runglall M, Tobouti P, Islam A, Hoyer LL, Naglik JR (2012). Evaluation of the role of *Candida albicans* agglutinin-like sequence (Als) proteins in human oral epithelial cell interactions. PLoS One.

[24] Xie J, Tao L, Nobile CJ, Tong Y, Guan G, Sun Y (2013). White-opaque switching in natural MTL a/α isolates of *Candida albicans*: evolutionary implications for roles in host adaptation, pathogenesis, and sex. PLoS Biol.

[25] Ray A, Dittel BN (2010). Isolation of mouse peritoneal cavity cells. J Vis Exp.

[26] Zhang Q, Tao L, Guan G, Yue H, Liang W, Cao C (2016). Regulation of filamentation in the human fungal pathogen *Candida tropicalis*. Mol Microbiol.

[27] Negri M, Botelho C, Silva S, Lopes LM, Henriques M, Azeredo J, Oliveira R (2011). An *in vitro* evaluation of *Candida tropicalis* infectivity using human cell monolayers. J Med Microbiol.

[28] Zuza-Alves DL, de Medeiros SSTQ, de Souza LBFC, Silva-Rocha WP, Francisco EC, de Araújo MC (2016). Evaluation of virulence factors in vitro, resistance to osmotic stress and antifungal susceptibility of *Candida tropicalis* isolated from the coastal environment of Northeast Brazil. Front Microbiol.

[29] da Silva EM, Kischkel B, Shinobu-Mesquita CS, Bonfim-Mendonça PS, Mansano ES, da Silva MA, et al. γ-irradiation from radiotherapy improves the virulence potential of Candida tropicalis. Future Microbiol. 2017; doi:10.2217/fmb-2017-0137. 10.2217/fmb-2017-0137 29110510

